# Transcriptome assembly and expression profiling of molecular responses to cadmium toxicity in hepatopancreas of the freshwater crab *Sinopotamon henanense*

**DOI:** 10.1038/srep19405

**Published:** 2016-01-20

**Authors:** Min Sun, Yi Ting Li, Yang Liu, Shao Chin Lee, Lan Wang

**Affiliations:** 1 School of Life Science, Shanxi University, Taiyuan 030006, China

## Abstract

Cadmium (Cd) pollution is a serious global problem, which causes irreversible toxic effects on animals. Freshwater crab, *Sinopotamon henanense*, is a useful environmental indicator since it is widely distributed in benthic habitats whereby it tends to accumulate Cd and other toxicants. However, its molecular responses to Cd toxicity remain unclear. In this study, we performed transcriptome sequencing and gene expression analyses of its hepatopancreas with and without Cd treatments. A total of 7.78 G clean reads were obtained from the pooled samples, and 68,648 unigenes with an average size of 622 bp were assembled, in which 5,436 were metabolism-associated and 2,728 were stimulus response-associated that include 380 immunity-related unigenes. Expression profile analysis demonstrated that most genes involved in macromolecular metabolism, oxidative phosphorylation, detoxification and anti-oxidant defense were up-regulated by Cd exposure, whereas immunity-related genes were down-regulated, except the genes involved in phagocytosis were up-regulated. The current data indicate that Cd exposure alters gene expressions in a concentration-dependent manner. Therefore, our results provide the first comprehensive *S.henanense* transcriptome dataset, which is useful for biological and ecotoxicological studies on this crab and its related species at molecular level, and some key Cd-responsive genes may provide candidate biomarkers for monitoring aquatic pollution by heavy metals.

Heavy metal contamination in aquatic environments, particularly in freshwater systems, has posed severe risks to organisms^1^,^2^,^3^,^4^,^5^. Cadmium (Cd), a non-essential but toxic heavy metal, is a worldwide pollutant in aquatic systems^3^,^4^,^5^,^6^. Since Cd has a long biological half-life, its toxic effects can be accumulated and bio-amplified through the food web^3^,^4^,^7^,^8^,^9^,^10^. Otherwise, Cd can induce morphological deformities, biochemical alterations and physiological dysfunctions, which lead to irreversible damage and even death^2^,^3^,^5^,^6^,^11^,^12^,^13^. Studies on its toxicity mechanisms have shown that Cd causes the elevation and accumulation of reactive oxygen species (ROS)^14^,^15^, which results in numerous injuries of cellular structures and biological functions, such as oxidative damage, protein destruction, DNA mutation, cellular membrane lipid peroxidation, cellular calcium homeostasis change, and cell junction alteration^5^,^6^,^8^,^11^,^12^,^13^,^14^,^15^.

Crustaceans are sensitive to heavy metal pollution, and crabs have been considered as suitable bioindicators^5^,^6^,^9^,^10^,^16^,^17^,^18^,^19^,^20^,^21^. Among them, freshwater crab, *Sinopotamon henanense*, is an important representative species of decapod crustaceans. Because it lives in the sediments of aquatic environments and is directly exposed to heavy metals, the crab has been confirmed to accumulate Cd in its main organs such as hepatopancreas, gill, gonad, and hemocytes^11^,^12^,^17^,^18^,^19^,^20^,^21^. In crustaceans, hepatopancreas is generally thought to be a key target organ for heavy metal toxicity and other environmental stresses^17^,^18^,^19^,^20^,^22^,^23^,^24^,^25^. Besides its functions in digestion and metabolism^19^,^20^,^26^,^27^,^28^, hepatopancreas also plays an important role in response to environmental stresses^17^,^18^,^19^,^20^,^24^,^25^,^29^. Previous studies have demonstrated that crabs have a compensatory metabolism with an increase of protease and aminotransferase activities of hepatopancreas and muscle in response to energy stress by acute Cd exposure^19^. There is also abundant evidence that activities of superoxide dismutase (SOD), catalase (CAT) and glutathione peroxidase (GPx) in hepatopancreas are significantly increased by Cd, which is assumed to remove the ROS and to resist oxidative damage^17^,^18^,^19^,^20^,^24^,^25^. Liu *et al.* demonstrated that low concentrations of Cd increased the inner membrane potential of mitochondria, and elevated activities of enzymes including succinate dehydrogenase (SDH), nicotinamide adenine dinucleotide oxidase and Ca^2+^ -ATPase^17^. Moreover, following exposure to high concentrations of Cd, some ultrastructural changes, such as chromatin condensation, nucleus and mitochondria swelling, membrane disruption, and cristae disappearance, were observed in the hepatopancreas^17^. Meanwhile, phagocytosis of hyaline cells was enhanced following Cd exposure, which might be required to remove the damaged macromolecules and cells^11^,^17^.

Recently, *glutathione S-transferases* (*GST*)^30^,^31^, *myeloid differentiation factor 88*^32^, and *toll like receptor* (*TOLL*)^33^ have been cloned from the hepatopancreas of *Macrobrachium rosenbergii*^30^, *Eriocheir sinensis*^22^,^31^ and *Litopenaeus vannamei*^32^,^33^. And, the Cd-induced gene expression alterations in *Procambarus clarkii* and *Palaemonetes pugio*^34^,^35^ as well as metabolic and transcriptional changes in *Daphnia magna*^5^,^6^ have been reported. However, the genome-wide molecular response to Cd toxicity remains unknown in crabs. The RNA sequencing approach and *de novo* assembly provide useful technology for transcriptome profiling in animals without any genomic information^5^,^6^,^22^,^23^,^25^,^26^,^27^,^28^,^36^,^37^,^38^,^39^,^40^,^41^. This state-of art technology allows us to perform transcriptomic analysis in the freshwater crab, *Sinopotamon henanense*, and thereby to reveal how the animals respond to Cd toxicity at the molecular level. In this study, we adopted a mixed sampling strategy to obtain the crab transcriptome database, and analyzed differential gene expression profiles of hepatopancreas samples treated with different concentrations of Cd. The main objectives were: (1) to construct the transcriptome dataset and annotate the generated genes; (2) to perform differentially expressed gene (DEG) analysis to identify genes responsive to Cd exposure; and (3) to characterize the Cd-altered biological processes and pathways and to reveal their association with metabolism, detoxification, immune response and other major biological functions.

## Results

### RNA sequencing and *de novo* transcriptome assembly

To obtain comprehensive transcriptome of the freshwater crab hepatopancreas, a cDNA library was constructed and sequenced from the pooled hepatopancreas samples of 12 individual crabs from the control without Cd treatment and three treatment groups with different Cd concentrations (7.25, 14.5 and 29.0 mg/L) (n = 3 in each group). A total of 77,799,168 (about 7.78 G) clean reads representing 7,001,925,120 clean nucleotides (nt) were produced. The average Q20 percentage and GC content were 97.06% and 50.22%, respectively (Table 1). *De novo* sequence assembly generated 180,318 contigs with a mean length of 279 bp and a total nucleotide length of 50,226,318 nt (Table 1). Among these contigs, 116,948 (64.86%) were smaller than 100–200 bp, 43,620 (24.2%) were between 200–500 bp, and 19,750 (10.95%) were longer than 500 bp (Supplementary Fig. S1a). Then the contigs were further assembled into 68,648 unigenes, varying from 200 to 14,143 bp with an average size of 622 bp, and 54,798 unigenes were singletons. In these unigenes, 63.55% were 100–500 bp and 36.45% were greater than 500 bp in size. In the latter group, 7.46% unigenes were longer than 1 kb and 4.29% were longer than 2 kb (Table 1 and Supplementary Fig. S1a).

### Functional annotation and classification

To search for the translation frame and the conserved protein domains of distinct unigenes, we performed BLAST against the NCBI non-redundant (nr) database, the Swiss-Prot, the Kyoto Encyclopedia of Genes and Genomes (KEGG), and the Clusters of Orthologous Groups (COG) database, with a cut-off E-value of 10^−5^. Their protein coding domains were predicted from 80.1% unigenes, while others were too short for the meaningful matches. A total of 26,625 unigenes (38.8%) were successfully annotated via BLASTx, and the returned data were above the cut-off E-value (Table 1). The remaining unigenes were further processed through the ESTscan analysis, and only 5,835 unigenes (8.5%) could be annotated, whereas up to 52.7% unigenes failed in matching to any known genes in public databases (Table 1 and Supplementary Fig. S1b). In the annotated unigenes, a total of 23,507 unigenes could be aligned to the sequences in the NCBI Nr database, and 41.08% of them had an E-value <1E-30 (Supplementary Fig. S2b), in which about 68% of the annotated unigenes belonged to crustacean species (Supplementary Fig. S2a). However, due to a lack of genome and EST information for *S. henanense*, less than a quarter of these aligned unigenes had 60% or more identity with those in the available databases (Supplementary Fig. S2c). Similarly, up to 84.2% and 71.6% of total unigenes failed in the Nt database and Swiss-Prot database, respectively (Table 1).

**Figure 1:**
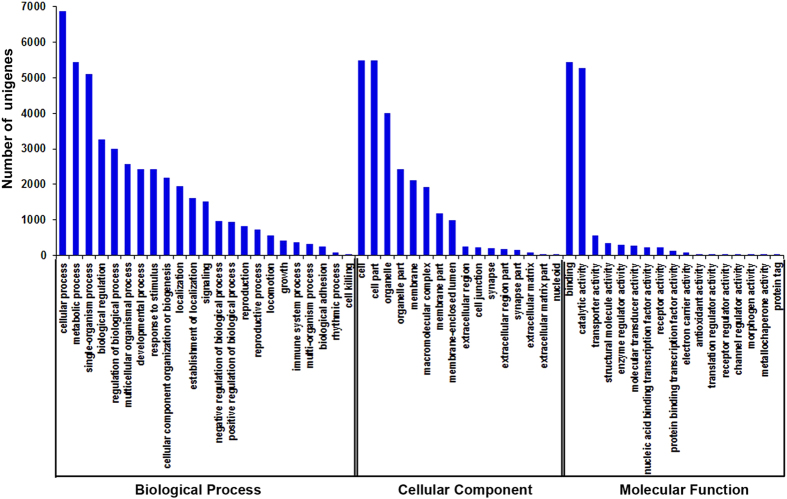
Gene Ontology (GO) categorization for assembled unigenes of the transcriptome. Each annotated sequence was assigned at least one GO term in three main categories (biological process, cellular component and molecular function), and 56 subcategories. The x-axis represents the GO term; the y-axis denotes the number of unigenes.

To characterize the transcriptome, the assembled unigenes were annotated by the COG database, by which 20,444 unigenes with significant homology were classified into 25 COG clusters (Supplementary Fig. S3). Among the functional clusters, “general function prediction” was the largest cluster that had 3,455 unigenes (41.14%), and “extracellular structures” (0.08%) was the smallest one. Obviously, the metabolism-associated clusters, such as “amino acid transport and metabolism”, “nucleotide transport and metabolism”, “carbohydrate transport and metabolism”, and “energy production and conversion”, were dominant. To analyze the functions of these unigenes, Gene Ontology (GO) assignments were performed. Based on sequence homology, 10,675 unigenes were categorized into 56 terms belonging to three major functional categories that include biological processes (23 terms), cellular components (16 terms), and molecular functions (17 terms) (Fig. 1). In these categories, terms of “metabolic process”, “cellular process”, “cell”, “cell part”, “binding” and “catalytic activity” had the largest number of unigenes that were annotated. Moreover, the terms of “response to stimulus”, “biological regulation” and “immune system process” had comparatively high percentages within the biological process category. The terms of “organelle”, “macromolecular complex” and “cell junction” were highly presented in the cellular component category. The terms of “transporter activity”, “enzyme regulator activity”, “molecular transducer activity”, “electron carrier activity” and “antioxidant activity” had higher percentages than other terms in the molecular function category. However, genes from the terms of “cell killing”, “nucleoid”, and “protein tag” had the lowest percentages in the three main categories, respectively (Fig. 1). Furthermore, to analyze the involved signal pathways, the unigenes were annotated to the KEGG database. A number of 17,169 unigenes were assigned to 258 KEGG pathways (Table 2). Among them, metabolic pathway, a significantly enriched pathway, had the most unigenes (2,470; 4.39%). Moreover, several pathways including regulation of actin cytoskeleton (879 unigenes), Huntington’s disease (507 unigenes), Salmonella infection (487 unigenes), Fc gamma R-mediated phagocytosis (436 unigenes), MAPK signaling pathway (346 unigenes), ABC transporters (293 unigenes) and toll-like receptor signaling pathway (83 unigenes) had relatively high percentages (Table 2). These annotations are useful to identify functional genes and specific biological processes in the crab hepatopancreas.

### Analysis of gene expression profiles and identification of DEGs responsive to Cd toxicity

To analyze gene expression differences, four expression libraries were respectively constructed from the control and the three Cd-treated groups (7.25, 14.5 and 29.0 mg/L) (n =3 in each group). Due to the fact that the pooling strategy might mask variations among these samples^35^,^40^,^41^, we performed four expression profile sequencing individually, and compared these profiles with each other. After illumina sequencing and removing the low quality tags, 5.59 M, 5.86 M, 5.87 M and 5.93 M clean tags were produced, and 197, 649, 103, 736, 97, 902 and 98, 672 distinct clean tags were obtained from the four libraries, respectively. As shown in Supplementary Table S1, more than 97% raw tags in each library are clean tags. Using the 68,648 unigene sequences from RNA-seq based transcriptome analysis as the reference database, 29,763 (43.36%), 22,272 (32.44%), 21,448 (31.24%) and 22,257 (32.42%) tags from the control and Cd-treated groups were mapped to the reference genes, respectively. The quantity and quality of the four expression profiles were analyzed in Supplementary Table S1 and Fig. S4, which indicated that despite the above differences, the quality of these sequencing data might be high enough to permit the following analyses.

**Figure 2:**
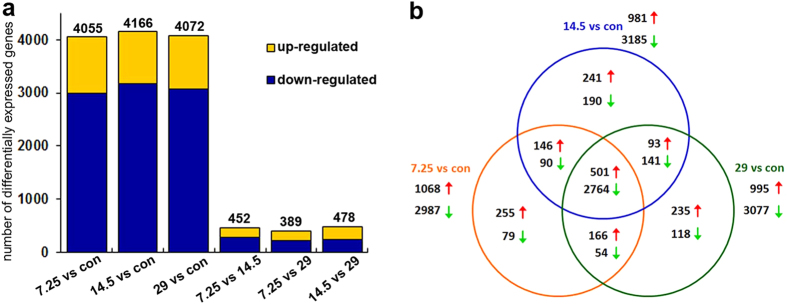
Analysis of DEGs following Cd exposure in the hepatopancreas. (**a**) Numbers of DEGs in each comparison. The number above each column shows the quantity of total DEGs. Up- and down-regulated unigenes are shown in yellow and blue, respectively. The x-axis shows six comparisons. The y-axis represents the total number of DEGs. (**b**) Venn diagrams of DEGs among groups with and without Cd treatment. This shows the numbers of up- or down-regulated unigenes from multiple comparisons among the four groups. Red upward arrows: up-regulated unigenes. Green downward arrows: down-regulated unigenes.

To screen responsive genes, we calculated and compared expression levels between the four groups (the control and the 7.25, 14.5 and 29.0 mg/L Cd-treated groups), respectively, and thereby identified DEGs with each other (Fig. 2). All DEGs with the absolute value of log2 Ratio ≥ 1 and the false discovery rate (FDR ≤ 0.001) were listed in Supplementary Tables S2,S3,S4-S5. As shown in Fig. 2, there are a total of 4,055 DEGs between the 7.25 mg/L group and the control, in which 1,068 and 2,987 unigenes are up-regulated and down-regulated respectively. Between the 14.5 mg/L group and the control, there exist a total of 4,166 DEGs, in which 981 unigenes and 3,185 unigenes are up-regulated and down-regulated respectively. Comparing 29.0 mg/L group with the control, there are a total of 4,072 DEGs, in which 995 and 3,077 unigenes are up-regulated and down-regulated respectively. In all of the appeared DEGs between the treatment and control groups, a total of 3,265 DEGs were commonly detected in all the treatment groups, in which 501 and 2,764 unigenes were found to be up-regulated and down-regulated respectively (Fig. 2 and Supplementary Tables S2,S4). Additionally, many DEGs had a similar expression tendency after different concentrations of Cd exposure. As shown in Fig. 2 and Supplementary Tables S3, S5, there are 175 and 277 up- and down-regulated unigenes respectively in the 7.25 mg/L group vs the 14.5 mg/L group, there are 168 and 221 up- and down-regulated unigenes respectively in the 7.25 mg/L group vs the 29.0 mg/L group, and there exist 231 and 247 up- and down-regulated unigenes respectively in the 14.5 mg/L group vs the 29.0 mg/L group. Significantly, these DEGs could be used to discover genes responsive to Cd stress in the crab, and thereby to identify some biomarkers for monitoring heavy metal pollution.

### The enriched gene functions and toxicity pathways

**Figure 3:**
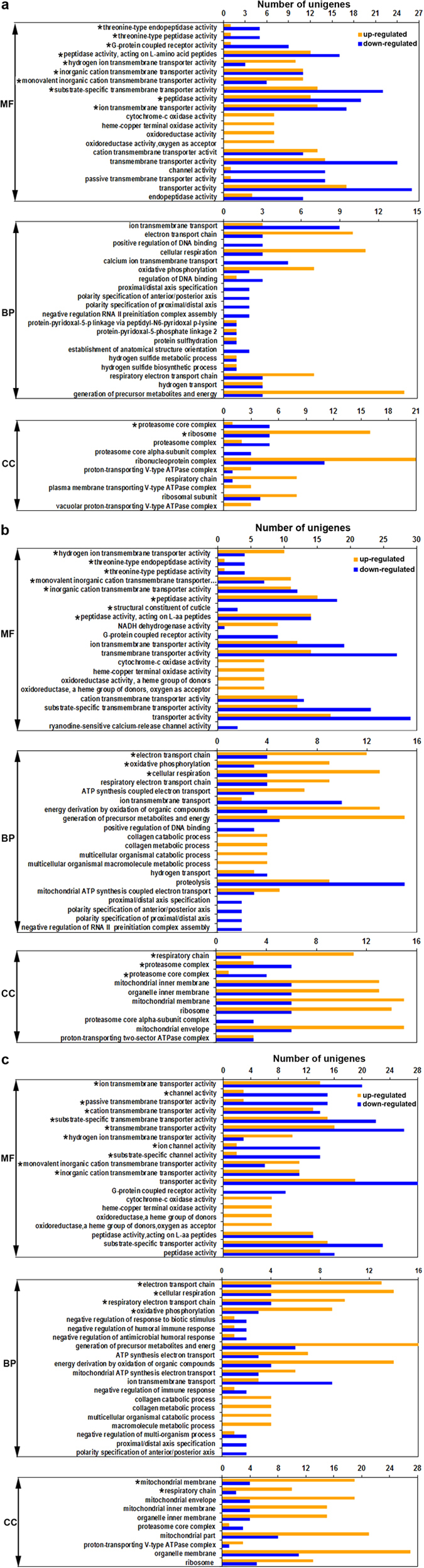
Up- and down-regulated unigenes of the top terms in comparisons between each Cd-treated group and the control. Top 20 molecular function terms, top 20 biological process terms and top 10 cellular component terms in three panels of comparisons between each Cd treatment groups and control groups. (**a**) 7.25 mg/L group vs control; (**b**) 14.5 mg/L group vs control; and (**c**) 29.0 mg/L group vs control. *p < 0.05.

To identify the biological function of DEGs, GO functional enrichment analyses were performed between the control and the three Cd treatment groups (Fig. 3 and Supplementary Tables S6,S7, Fig. S5). Significantly, most DEGs were enriched in metabolism and energy generation including “multicellular organismal macromolecule metabolic process”, “nucleic acid metabolic process”, “protein complex biogenesis”, “lipid metabolic process”, “respiratory electron transport chain”, “oxidative phosphorylation” and “ATP synthesis coupled electron transport”. Some other terms of DEGs were associated with stimulus response, which included detoxification and immune system, such as “response to chemical stimulus”, “response to oxidative stress”, “immune response” and “innate immune response” (Supplementary Tables S6,S7). To further specify the up- or down-regulated genes from each comparison, we analyzed top terms from the six comparisons between the treated three samples and the control. Figure 3 lists top 20 terms in molecular function category, top 20 terms in biological process category, and top 10 terms in cellular component category. Supplementary Fig. S5 shows top 10 terms of the three main categories between each other of the treatment groups. Overall, gene number of a term increased with the increase in Cd concentration, and the up-regulated unigenes outnumbered the down-regulated ones.

**Figure 4:**
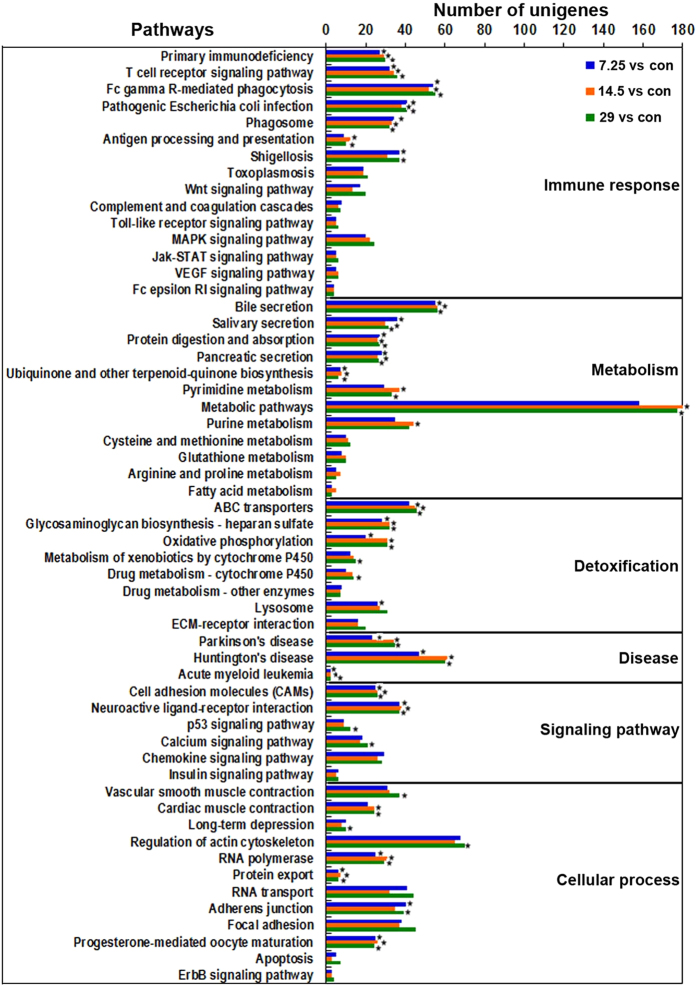
Distribution of DEGs in representative pathways responsive to Cd toxicity in comparisons between each Cd-treated groups and the control. KEGG enrichment analysis was performed to identify pathways responsive to different concentrations of Cd. *Q < 0.05.

To understand the Cd-affected pathways, DEGs were analyzed against KEGG database for pathway enrichment. Between each of the three treatment groups and the control, a total of 213, 214, and 215 pathways were enriched, in which 25, 27 and 32 were significantly enriched, respectively (Supplementary Table S8). As shown in Fig. 4, the maximum number of DEGs appears in metabolic pathways, and the involved macromolecular metabolism pathways include protein digestion and absorption, ubiquinone and other terpenoid-quinone biosynthesis, and pyrimidine metabolism. The significantly enriched detoxification-associated pathways include ABC transporters, metabolism of xenobiotics by cytochrome P450, and oxidative phosphorylation. And, many immunity-associated pathways, such as Fc gamma R-mediated phagocytosis, phagosome, primary immunodeficiency, toll-like receptor signaling, T-cell receptor signaling, Jak-STAT signaling, MAPK signaling, and VEGF signaling, are also enriched.

### Validation of expression profiles by RT-qPCR

**Figure 5:**
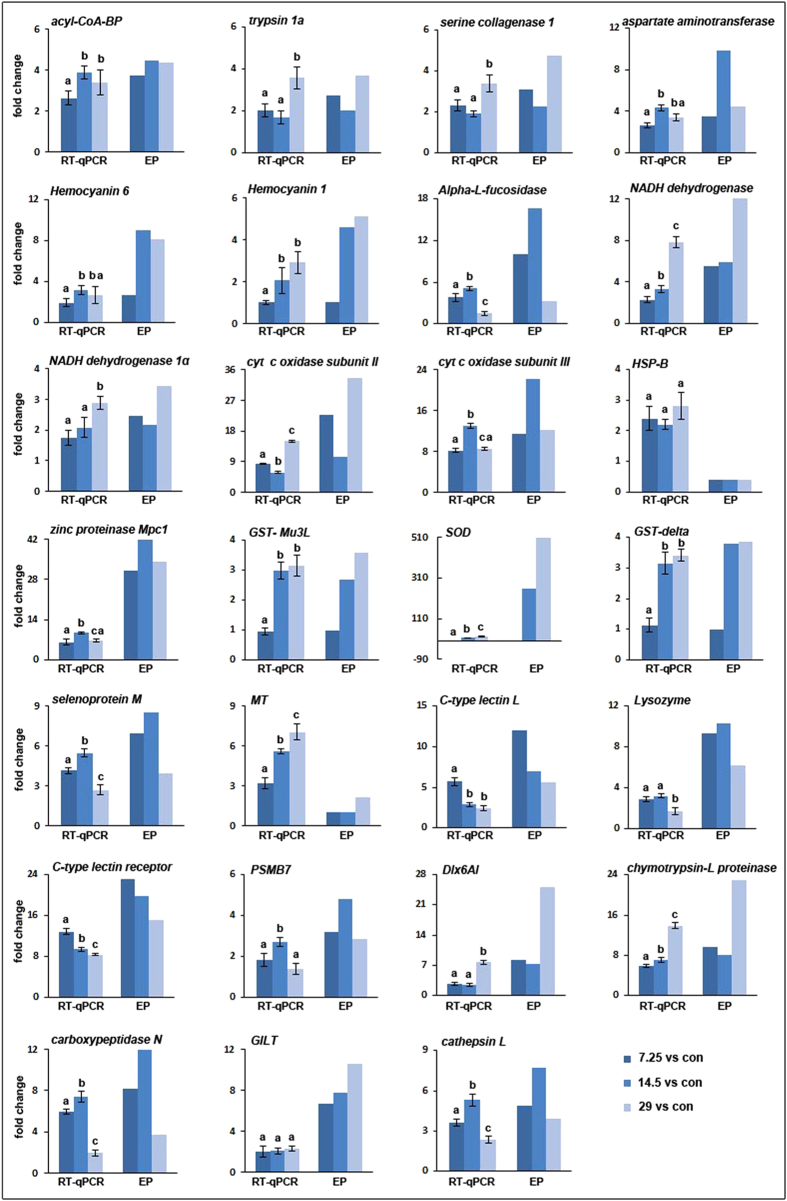
Comparison between RT-qPCR data and expression profiles. A total of 27 genes, such as acyl-CoA-binding protein (acyl-CoA-BP), trypsin 1a, serine collagenase 1, aspartate aminotransferase, hemocyanin subunit 6 (Hemocyanin 6), hemocyanin subunit 1 (Hemocyanin 1), Alpha-L-fucosidase, NADH dehydrogenase 5, NADH dehydrogenase 1α, cytochrome c oxidase subunit II (cyt c oxidase subunit II), cytochrome c oxidase subunit III (cyt c oxidase subunit III), heat shock protein binding (HSP-B), zinc proteinase Mpc1, glutathione S-transferase Mu 3-like (GST- Mu3L), superoxide dismutase (SOD), delta glutathione S-transferase (GST-delta), selenoprotein M, Metallothionein (MT), C-type lectin L, Lysozyme, C-type lectin receptor, proteasome subunit beta type-7-like (PSMB7), homeobox protein Dlx6a-like (Dlx6AL), chymotrypsin-like proteinase, carboxypeptidase N, gamma-interferon-inducible lysosomal thiol reductase (GILT), cathepsin L, were analyzed by RT-qPCR and comparized with expression profile (EP), respectively. Data are presented as mean fold changes (n = 4 crabs per treatment) ± S.E. Different letters above each column indicate significant differences among different groups (p < 0.05), using Mann-Whitney U method in the post hoc testing, after Kruskal-Wallis analysis in non-parametric method.

To confirm the expression profile data, we further examined relative expression levels of 27 interested genes, and quantified their relative expression folds between the control group and three different Cd-treated groups by RT-qPCR. A total of 16 crabs from the control group and three Cd treatment groups (n = 4 in each group) were used for each gene validation. In the analyzed 27 genes, up to 24 genes were detected to have similar fold changes to expression profile data, whereas inconsistent expression was observed only in three genes, such as *MT*, *SOD*, and *HSP-B*, in which the fold changes of *HSP-B* and *MT* detected by RT-qPCR were higher than expression profile, while the fold change of *SOD* was lower than expression profile (Fig. 5). These comparative analyses revealed more than 88.8% concordance rate between RT-qPCR analysis and expression profile data, validating the accuracy and reliability of the sequencing data.

## Discussion

Cadmium is one of common heavy metal pollutants^4^,^5^,^6^,^7^,^8^, but the molecular events triggered by Cd have remained largely unknown in the aquatic environmental indicator species of crabs due to the lack of genomic information. In this study, we used the target organ hepatopancreas of *S. henanense* to perform a genome-wide investigation by transcriptional sequencing and gene expression profile analysis, and confirmed the accuracy and reliability through RT-qPCR. This is the first transcriptomic analysis of crabs with relevance to Cd toxicity, and provides a potential biomarker database for monitoring aquatic environmental pollution by heavy metal in invertebrates.

### The first transcriptomic study in freshwater crab *S. henanense*

In this study, a total of 68,648 unigenes were assembled from the hepatopancreas of freshwater crab *S. henanense* either with or without Cd exposure (Table 1), which was the first transcriptomic study for the crab. Li *et al.* performed transcriptomic study in the hepatopancreas from *E. sinensis* challenged with microbes and obtained 70,300 assembled unigenes^22^. They detected 1,652 unigenes more than we did in our samples, which might reflect the difference in biological functions between the two species. In our transcriptome data, up to 61.2% of unigenes failed in BLAST annotation (Table 1 and Supplementary Fig. S2), which was likely due to limited information about the genomes or transcriptomes of the crab and its related species^22^,^26^,^27^,^28^. These unmatched unigenes might be candidates for novel gene discovery. Many unigenes were annotated in categories and pathways related to metabolism, detoxification and immunoresponse (Fig. 1, Table 2, and Supplementary Fig. S3), which might reflect the major physiological differences of hepatopancreas in crabs^17^,^18^,^19^,^20^,^22^,^26^,^27^,^28^.

### Cd exposure alters gene expressions in a concentration-dependent manner

Previously, Cd had been shown to cause tissue cell damages and alter redox balance in a concentration-dependent manner in the crab hepatopancreas^17^,^18^,^19^,^20^. In the current transcriptomic analysis, Cd was further confirmed to alter gene expression also in a concentration-dependent manner, and the number of DEGs increased along with the increment of Cd concentration (Fig. 2 and Supplementary Tables S2,S4). The samples treated with different concentrations of Cd (7.25, 14.5 and 29.0 mg/L) showed different DEG numbers in the same terms and pathways from GO and KEGG analyses (Figs 3,4, and Supplementary Fig. S5). There were much more down-regulated genes than up-regulated ones by comparing the three treatment groups with the control (Fig. 2 and Supplementary Tables S2–S5). This implied that gene expressions were largely inhibited by Cd, which might lead to impairments of biological functions. Obviously, the expression alteration significance of specific genes or pathways remains to be further investigated.

### Major biological associations in response to acute Cd stress in *S. henanense*

Metabolism is one of the major functions of hepatopancreas^19^,^20^,^26^,^27^,^28^. In this study, many genes and pathways involved in macromolecular metabolism and energy production were found to undergo expression changes in the hepatopancreas following Cd exposure (Figs 1,3,4,5, Table 2, and Supplementary Figs S2,S4). The finding is consistent with our previous biochemical studies that Cd increases the activities of metabolic enzymes, such as protease, alanine aminotransferase and aspartate aminotransferase, and enhances the mobilization of carbohydrate, protein and fat^17^,^18^,^19^,^20^. To our knowledge, major energy supply in the body is from oxidative phosphorylation in mitochondria. Liu *et al.* demonstrated that the functions of mitochondria were enhanced in the crab to generate energy to prevent the bodies from damages^17^. In keeping with this, in our expression profile analysis, terms or pathways involved in the mitochondrial respiratory chain such as oxidative phosphorylation, electron transport chain and ATP synthesis coupled electron transport were significantly enriched (Figs 3,4,5, and Supplementary Figs S3,S5, Table S8). The activities of key enzymes in the respiratory chain, such as cytochrome c oxidase (COX), SDH and Ca^2+^ -ATPase, were increased significantly, and the contents of cytochrome c (cyt c) and NADPH were elevated in the crabs after Cd treatment^17^,^19^,^20^. Similar to these observations, *COX* and *NADH dehydrogenase* were also revealed to be up-regulated by Cd (Fig. 5 and Supplementary Tables S2–S5). Thus, our current findings have identified some significant differentially expressed genes involved in metabolism, and provided molecular bases for studying response mechanism of metabolism under environmental stress.

Detoxification is another important function of hepatopancreas in crustaceans^17^,^18^,^24^,^30^,^31^. In previous reports, Cd exposure was revealed to promote oxidative damage due to the cellular concentration increase of ROS^14^,^15^. Enzymes involved in detoxification and anti-oxidative defense could remove excess ROS to reduce oxidative stress, which were extensively examined in crabs in the past decade. Increase in activities of SOD, CAT, GPX and GST in response to Cd were noted^11^,^12^,^17^,^18^,^19^,^20^,^21^. The redox regulation processes were significantly enriched in GO terms and pathways in the present study (Figs 3,4, and Supplementary Tables S2,S3,S6–S8), suggesting that the expression of large number of genes involved in anti-oxidant defense might be modified to protect cells against oxidative damages triggered by Cd. For example, the expression level of *SOD* was significantly increased with increasing concentration of Cd (Fig. 5 and Supplementary Tables S2,S4), confirming our previous results that the enzymatic activity of SOD was elevated after Cd exposure^17^,^18^. GST, a multifunctional enzyme superfamily, plays a critical anti-oxidative role in the detoxification and protection of organisms against oxidative stress^25^,^30^,^31^. In a previous report, we found that GST activity decreased initially then increased with increasing concentration of Cd^18^. Our expression profile demonstrated that the expression of *GST-theta1* and *GST-CL5572* was significantly down-regulated under 7.25 mg/L Cd exposure, while *GST-delta* and *GST-Mu3L* were up-regulated when 14.5 mg/L and 29 mg/L Cd exposure (Fig. 5 and Supplementary Tables S2,S4). Possibly, the increased GST activity with the higher concentration of Cd is due to high expression of *GST-delta* and *GST-Mu3L*. In *M. rosenbergii* challenged with *Vibrio*, *MrMuGST1* and *MrMuGST2* were down-regulated, while *MrMuGST3* and *MrMuGST4* were up-regulated^30^. In *E. sinensis*, *GST-delta* was significantly induced by bacterial challenge^22^,^31^. Additionally, cytochrome P450 enzyme (CYP450) is one of the critical detoxification enzymes, and is considered to be a biomarker in most aquatic animals^25^,^42^. In our transcriptome sequencing, 105 *CYP450* transcripts were assembled in total, but only 6 members of the gene family were differentially expressed when compared with the control: *CYP2L1* and *CYP379A1* were up-regulated, and *CYP3A4L*, *CYP6BQ13*, *CYP2L* and *CYP330A1* were down-regulated (Supplementary Fig. S6 and Tables S2,S4). Therefore, different types of responsive genes may function in different manners in response to environmental stress, and these molecules may be used as biomarkers to assess the toxic effects on animals by heavy metals in aquatic environments.

**Figure 6:**
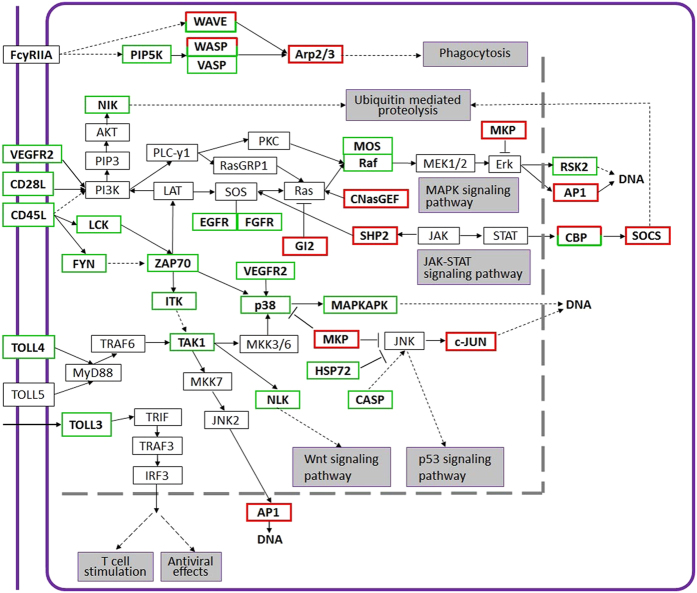
Changes of immunity-related DEGs and pathways after Cd exposure in the hepatopancreas. Red box: up-regulated unigene(s). Green box: down-regulated unigene(s). Box with both red and green: pathway includes unigene(s) up- and down-regulated, respectively.

The immune system is generally sensitive to environmental stresses including heavy metal toxicity. Like other invertebrates, crabs lack an adaptive immune system, but have innate immunity. Lectin, cathepsin L and other enzymes in lysosome have been reported to involve and enhance the function of phagocytosis^17^,^22^,^43^,^44^. In our expression profile analyses, *cathepsin L*, *lysozyme*, *c-type lectin*, *c-type lectin receptor* and *GILT* were revealed to be significantly up-regulated and enriched when crabs were exposed to Cd (Figs 4,5,6, and Supplementary Tables S2, S4, S8), suggesting that phagocytosis activity might be induced to remove and clear damaged cells and macromolecules^11^,^17^,^45^,^46^. In contrast, numbers of immune-related genes were down-regulated after Cd exposure (Fig. 6 and Supplementary Tables S2,S3,S8). The expression of *prophenoloxidase* and *acid phosphatase* (*ACP*) were significantly down-regulated (Supplementary Tables S2,S3), supporting previously biochemical observations that phenoloxidase activity was reduced by Cd and nickel in *Litopenaeus vannamei*^45^ and *Scylla serrata*^46^ respectively, and the activity of the ACP was decreased by Cu in the hepatopancreas of *M. rosenbergii*^47^. Moreover, as shown in Fig. 6, the immune-associated pathways such as VEGF signaling, toll-like receptor signaling, T-cell receptor signaling, Jak-STAT signaling, MAPK signaling are affected by Cd toxicity, and the expression of *VEGFR2*, *TOLL3*, *TOLL4*, etc. are suppressed. Thus, Cd might impair immunity by inhibiting the expression of immune-related genes. This is different from results that immunity-associated factors are up-regulated in crustaceans challenged with pathogens which trigger immunoresponses^22^,^23^,^32^,^33^.

In summary, we obtained 68,648 unigenes from the freshwater crab, *S. henanense* by transcriptomic sequencing and identified numerous Cd stress-associated DEGs and signal pathways by gene expression profiling. These data suggested that the crab might counteract the toxicity of Cd at the transcriptomic level by increasing expression levels of metabolism-associated, detoxification-related and anti-oxidative defense-related genes. Additionally, the immune functions might be impaired by Cd, whereas phagocytosis might be enhanced to remove the damaged macromolecules. Overall, this study provides the first informative reference dataset for future studies on global and specific response to environmental stresses (i.e. Cd) at the molecular level, and will facilitate gene discovery and biomarker identification in the crab and other decapod species.

## Methods

### Animals and treatments

Adult freshwater crabs of *S. henanense*, were purchased from the Dongan aquatic market in Taiyuan in Shanxi province, China. Prior to experiments, crabs were acclimated for 2 weeks in glass aquaria (50 cm × 30 cm × 25 cm) with 3–4 cm depth of dechlorinated tap water (dissolved oxygen 8.0–8.3 mg/L, pH 7.4) maintained at 20 ± 2 °C, and fed three times a week with commercial feed^11^,^12^,^17^,^18^,^19^,^20^,^21^ (ZhongShan Uni-President company, Guangzhou, China). Half of the aquaria were shielded by a black plastic to reduce disturbances and constant aeration was maintained. Aquaria were checked every day and dead animals were removed. Water was changed three times a week after aquaria were cleaned thoroughly. Only healthy adult crabs without any damage and pathological signs, and with a consistent weight of 21.57 ± 0.78 g were selected and used for the experiments. In previous studies, Cd caused significant changes in tissue structure and function of hepatopancreas at 14.5 mg/L Cd exposure for 48 h^11^,^17^,^18^,^19^. Thus, in this study, the crabs were randomly divided into four experimental groups with 5–8 individual crabs in each group and allocated to 0 mg/L (control), 7.25 mg/L, 14.5 mg/L and 29.0 mg/L Cd (Sigma, St. Louis, MO, USA) for 48 h, corresponding to 1/32, 1/16, 1/8 of the 96 h LC_50_, respectively^11^,^12^,^17^,^18^,^19^,^20^,^21^. During the experiments, crabs were not fed, and dead ones were immediately removed. At the end of exposure, the hepatopancreas of each crab from the control group and three Cd treatment groups (4 individual crabs in each group) was respectively sampled and weighed, and each hepatopancreas sample was divided into two aliquots and immediately stored in liquid nitrogen for the following experiments. The Animal Care Committee at Shanxi University approved all animal experiments. The methods in this research were carried out at Shanxi University in accordance with the approved guidelines.

### RNA extraction and transcriptome sequencing

To perform transcriptome and expression profile analyses, four groups of equal amount hepatopancreas samples were respectively combined by three individual hepatopancreas aliquots from the control group and three Cd treatment groups (n = 3 in each group), and their total RNAs were extracted by using Trizol Reagent (Takara, Shiga, Japan). The quality and integrity of these total RNA samples were determined by an Agilent 2100 Bioanalyzer (Agilent Technologies, Santa Clara, CA, USA), and their concentrations were between 2660 ng/μL and 3240 ng/μL with the RNA integrity number ranging from 4.2 to 5.0. These RNA samples passed the quality tests and were used for further process. The extracted total RNA samples were incubated in DNAse I (Ambion, Austin, TX, USA) for 1 h at 37 °C to remove genomic DNA, and mRNAs were purified from these total RNAs using oligo (dT) beads and Oligotex mRNA Kits (Qiagen, Valencia, CA, USA). Moreover, the four group RNA samples were equally divided into two portions, and their one portion were respectively stored and used to construct 4 separate cDNA libraries of the control group and three Cd treatment groups for expression profile sequencing, and another portion were combined into a pooled sample for transcriptome sequencing. Subsequently, the pooled mRNAs were disrupted into short fragments, and used as templates to synthesize first-stranded cDNA following a random hexamer-primer and reverse transcriptase (Life Technologies, Gaithersburg, MD, USA). Second-strand cDNAs were synthesized using RNase H and DNA polymerase I. Moreover, the double strand cDNAs were purified and modified by sequencing adaptors, and, the suitable fragments were selected by gel purification, and enriched by PCR amplification to create a cDNA library. Finally, the library was subjected to sequencing using the Illumina HiSeq^TM^ 2000 sequencing platform (Illumina, San Diego, CA, USA).

### *De novo* assembly and annotation of illumina sequencing data

The raw sequences were collected by removing adaptor sequences, reads with ambiguous ‘N’ nucleotides (the ratio of ‘N’ ≥ 5%) and low quality sequences. Then, the remaining clean reads were *de novo* assembled by Trinity software, a short-read assembly program for *de novo* transcriptome assembly without reference genome^48^. Trinity firstly combined clean reads to form longer fragments, contigs, and then assembled contigs into unigenes. Finally, all of the clean reads were pooled together and assembled to form the global transcriptome data of *S. henanense*.

For annotation, sequencing data were aligned using BLASTx alignment (E-value cut-off of 10^−5^) with protein databases, including NCBI Nr database, Swiss-Prot (http://www.ebi.ac.uk/uniprot/), COG (http://www.ncbi.nlm.nih.gov/COG/) and KEGG (http://www.genome.jp/kegg/) database. The sequence direction of unigenes was decided by the best alignment. If the sequence annotation of different databases was in conflict, a priority order of alignments of Nr, Swiss-Prot, KEGG and COG databases was followed to decide sequence direction of the unigenes. Furthermore, ESTscan software was used to predict the coding regions and decide its sequence direction when the unigene could not align in any of the above databases^49^. Based on Nr annotation, BLAST2GO program^50^ was used for GO analysis (http://www.geneontology.org/). Further, COG classification and signal pathway annotation of unigenes was performed by BLASTx searching against the COG database and KEGG database.

### Differential expression library construction, gene expression profile sequencing and annotation

The separately stored 4 RNA samples from the control group and three Cd treatment groups (n = 3 in each group) were respectively used to isolate the mRNAs by oligo (dT) beads and Oligotex mRNA Kits (Qiagen, Valencia, CA, USA), and the isolated mRNAs were used to construct 4 differential cDNA libraries in parallel with Digital Gene Expression (DGE) tag profile kit (Illumina, San Diego, CA, USA), according to the manufacturer’s protocol (Version 2.1B). In brief, each isolated mRNA sample was respectively enriched by oligo-dT magnetic beads, and prepared for synthesizing double stranded cDNA. Subsequently, the bead-bound cDNA was digested with *Nla*III and added Illumina adapter 1, and then digested with *Mme*I and added Illumina adapter 2 to create the sequencing library. After amplification by PCR, cDNA tag library was purified and checked. After the 4 differential cDNA tag libraries were prepared, they were respectively denatured, fixed onto the illumina proprietary sequencing chip (flow cell) for *in situ* amplification, and finally performed sequencing by Illumina sequencing platform (Illumina, San Diego, CA, USA).

The sequenced raw data included the adaptor as well as a few low-quality sequences and several types of impurities. Raw data were transformed into clean tags after certain steps of data-processing, including removing the adaptor sequence, and removing low-quality tags (tags with unknown nucleotides ‘N’ or only one copy), too long or too short tags and empty reads with adaptor sequence only. For annotation, all clean tags were mapped to our transcriptomic reference database generated by this studies’ sequencing, allowing no more than 1 bp mismatch^48^,^51^. Clean tags mapped to reference sequences after multiple genes were filtered. Remainder clean tags were designed as unambiguous clean tags. The number of unambiguous clean tags for each gene was calculated and then normalized to the number of transcripts per million clean tags^52^.

### Analysis of DEGs

The expression level of each unigene from the four separate libraries was calculated and normalized to reads per kilo base per million (RPKM)^51^,^53^ according to the number of expression tags. The differences in gene expression abundance between the control and treatment groups were counted and statistically analyzed based on the MARS model (MA-plot-based method with random sampling model) using the DEGseq package^54^. To identify the DEGs, FDR ≤ 0.001 and two-fold change (log2 Ratio) ≥ 1 or ≤ −1 were set to be the threshold for judging the significance of gene expression differences^55^. The four gene expression profiles were compared with each other, and then all DEGs in each comparison were carried on the GO functional and KEGG pathway enrichment analysis using GO database and KEGG database. The DEGs mapped to the KEGG database were screened and analyzed by MapMan software.

### RT-qPCR validation

The stored hepatopancreas aliquot from each crab of the control group and the three Cd treatment groups (4 individual crabs in each group) was respectively used to extract the total RNA sample as described above. The quality and quantity of total RNA for RT-qPCR analysis were measured by BioSpectrometer fluorescence (Eppendorf, Hamburg, Germany). And, the RNA samples that showed A_260_/A_280_ ratio from 1.8 to 2.0 with the concentrations between 500 ng/μL and 900 ng/μL were used for the validation. The first strand cDNA was synthesized from 2 μg of total RNA using cDNA synthesis kit (Takara, Shiga, Japan). The RT-qPCR was performed in an ABI 7500 real-time detection system (Applied Biosystems, Foster City, CA, USA) using SYBR Premix Ex Taq II (Takara, Shiga, Japan) according to the manufacturer’s instructions and all experiments were performed in triplicates including NTC. After detecting program of each PCR, melting curve analysis was applied to all reactions. Only primers with single peak were used for further detection. The RT-qPCR primers were designed based on the transcriptome sequences using Primer 5 software^56^,^57^,^58^ and listed in Supplementary Table S9. A range of series dilution of cDNA (10^n^-fold) was used to create the five-point standard curve with the correlation coefficient (R^2^) of each curve greater than 0.99. The equation (E = 10^(1/-slope)^ −1) was used to calculate the RT-qPCR amplification efficiency (E)^56^,^57^,^58^. Primers with the E values between 95% and 105% were used in this study (Supplementary Table S9). Five reference genes of 18S *rRNA, Rpl38, GAPDH, Rpl13* and *Rpl44* were assessed by NormFinder software^59^, which showed that their expression stabilities were ranked as *Rpl38* > *Rpl44* > *Rpl13* > 18S *rRNA* > *GAPDH*. According to the assessment, two most stable reference genes (*Rpl38 and Rpl44*) were used to normalize RT-qPCR data^59^. Relative expression levels of the detected target genes from different samples were calculated according to standard curves generated from both the target gene and internal reference gene. Four biological replicates were used in each group. These experimental data were statistically analyzed by a non-parametric method using the computer software package SPSS v21.0. (IBM, Chicago, IL,USA) as described^60^,^61^, and presented as mean (n = 4) ± S.E. Kruskal-Wallis test and Mann-Whitney U test were carried out to analyze the differences among the different treatment groups^61^. A p-value < 0.05 was considered as statistically significant.

## Additional Information

**How to cite this article**: Sun, M. *et al.* Transcriptome assembly and expression profiling of molecular responses to cadmium toxicity in hepatopancreas of the freshwater crab *Sinopotamon henanense*. *Sci. Rep.* **6**, 19405; doi: 10.1038/srep19405 (2016).

## Supplementary Material

Supplementary Information

Supplementary Table S2

Supplementary Table S3

Supplementary Table S4

Supplementary Table S5

Supplementary Table S6

Supplementary Table S7

Supplementary Table S8

## Figures and Tables

**Table 1:** Summary of transcriptome characterization of *Sinopotamon henanense*.

Total clean reads	77,799,168
Total nucleotides (nt)	7,001,925,120
Q20 percentage	97.06%,
GC percentage	50.22%
Total number of contigs	180,318
Total length of contigs (nt)	50,226,318
Average length of contig (bp)	279
Total number of unigenes	68,648
Average length of unigenes (bp)	622
Number of singletons	54,798
NR annotation (%)	23,507 (88.3%)
NT annotation (%)	10,853 (40.8%)
Swiss-Prot annotation (%)	19,471 (73.1%)
KEGG annotation (%)	17,169 (64.5%)
COG annotation (%)	8,399 (31.5%)
GO annotation (%)	10,675 (40.1%)
Total (%)	26,625 (38.8%)

**Table 2:** KEGG pathway annotation of the transcriptomic unigenes.

Pathway ID	Pathway	Number of unigenes (17169)
ko01100	Metabolic pathways	2470 (14.39%)
ko04810	Regulation of actin cytoskeleton	879 (5.12%)
ko05146	Amoebiasis	681 (3.97%)
ko03013	RNA transport	628 (3.66%)
ko04510	Focal adhesion	614 (3.58%)
ko03040	Spliceosome	584 (3.4%)
ko05110	Vibrio cholerae infection	570 (3.32%)
ko05200	Pathways in cancer	550 (3.2%)
ko05016	Huntington’s disease	507 (2.95%)
ko05132	Salmonella infection	487 (2.84%)
ko05169	Epstein-Barr virus infection	480 (2.8%)
ko00230	Purine metabolism	479 (2.79%)
ko04120	Ubiquitin mediated proteolysis	441 (2.57%)
ko04666	Fc gamma R-mediated phagocytosis	436 (2.54%)
ko04520	Adherens junction	432 (2.52%)
ko04062	Chemokine signaling pathway	426 (2.48%)
ko04144	Endocytosis	423 (2.46%)
ko05100	Bacterial invasion of epithelial cells	405 (2.36%)
ko05131	Shigellosis	403 (2.35%)
ko03015	mRNA surveillance pathway	403 (2.35%)
ko05168	Herpes simplex infection	395 (2.3%)
ko04142	Lysosome	374 (2.18%)
ko04141	Protein processing in endoplasmic reticulum	374 (2.18%)
ko05202	Transcriptional misregulation in cancer	363 (2.11%)
ko05166	HTLV-I infection	362 (2.11%)
ko05130	Pathogenic Escherichia coli infection	357 (2.08%)
ko04530	Tight junction	351 (2.04%)
ko04270	Vascular smooth muscle contraction	346 (2.02%)
ko04010	MAPK signaling pathway	346 (2.02%)
ko04976	Bile secretion	341 (1.99%)
ko00240	Pyrimidine metabolism	337 (1.96%)
ko00310	Lysine degradation	329 (1.92%)
ko04110	Cell cycle	324 (1.89%)
ko04080	Neuroactive ligand-receptor interaction	319 (1.86%)
ko04512	ECM-receptor interaction	315 (1.83%)
ko05414	Dilated cardiomyopathy	307 (1.79%)
ko05164	Influenza A	304 (1.77%)
ko05410	Hypertrophic cardiomyopathy (HCM)	302 (1.76%)
ko03008	Ribosome biogenesis in eukaryotes	300 (1.75%)
ko04145	Phagosome	293 (1.71%)
ko02010	ABC transporters	293 (1.71%)
ko04962	Vasopressin-regulated water reabsorption	280 (1.63%)
ko05010	Alzheimer’s disease	267 (1.56%)
ko05014	Amyotrophic lateral sclerosis (ALS)	264 (1.54%)

Pathways listed only with high percentage (more than 1.5%) of annotated unigenes.
