# Supplementary material for: Transcriptome assembly and expression profiling of molecular responses to cadmium toxicity in hepatopancreas of the freshwater crab Sinopotamon henanense
Source: Sci Rep. 2016 Jan 20;6:19405. doi: 10.1038/srep19405 (PMC4726346; doi:10.1038/srep19405)
Supplement: Supplementary Information [file srep19405-s1.doc]

**Transcriptome assembly and expression profiling of molecular responses to cadmium toxicity in hepatopancreas of the freshwater crab *Sinopotamon henanense***

Min Sun^§^, Yi Ting Li^§^, Yang Liu, Shao Chin Lee, Lan Wang^*^

School of Life Science, Shanxi University, Taiyuan 030006, China.

^§^These authors contributed equally to this work.

^*^Correspondence author

Prof. Lan Wang

School of Life Science, Shanxi University, No. 92 Wucheng Road,

Taiyuan 030006, Shanxi, China.

E-mail: [lanwang.sxu@qq.com](mailto:lanwang.sxu@qq.com)

Tel/Fax: +86-351-7011429

**Supplementary information includes:**

**Supplementary Figures S1-S6 and Supplementary Tables S1-S9.**

**Supplementary figure legends**

**Supplementary Figure S1. The length distribution of sequences.**

**Supplementary Figure S2. Analysis of assembled unigenes of the transcriptome annotated using Nr databas**e.

**Supplementary Figure S3. Clusters of Orthologous Groups annotation of the transcriptome.**

**Supplementary Figure S4. Evaluation and comparisons of the *de novo* assembled sequences of the four gene expression profiles.**

**Supplementary Figure S5. Up- and down-regulated unigenes of the enriched GO terms among Cd-treated groups.**

**Supplementary Figure S6. Identification of unigenes associated with the detoxification pathways in the hepatopancreas.**

**Supplementary figures**

**
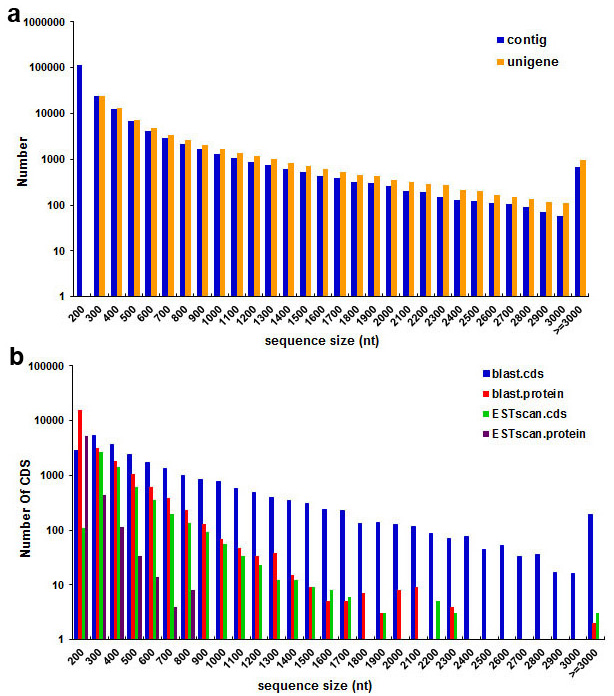
**

**Supplementary Figure S1.** (a) The length distribution of the assembled contigs and unigenes. (b) The length distribution of coding domain of assembled transcripts predicted by blast and ESTscan.


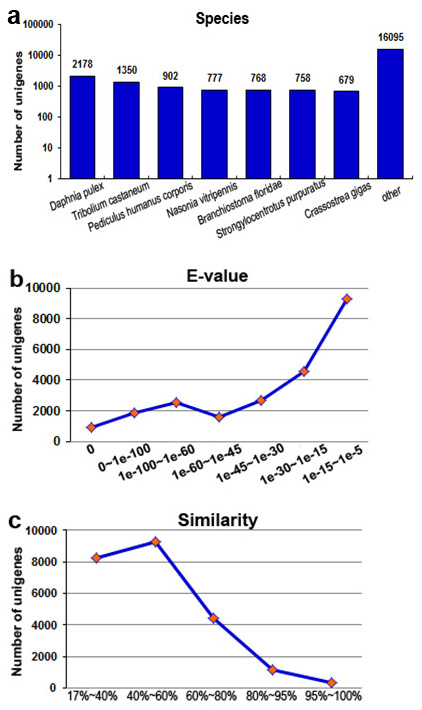


**Supplementary Figure S2. Analysis of assembled unigenes of the transcriptome annotated using Nr databas**e. Species, e-value and similarity distributions of the assembled unigenes against database are shown: (a) Species distribution of annotated unigenes, (b) E-value distribution of annotated unigenes, (c) Similarity distribution of annotated unigenes.


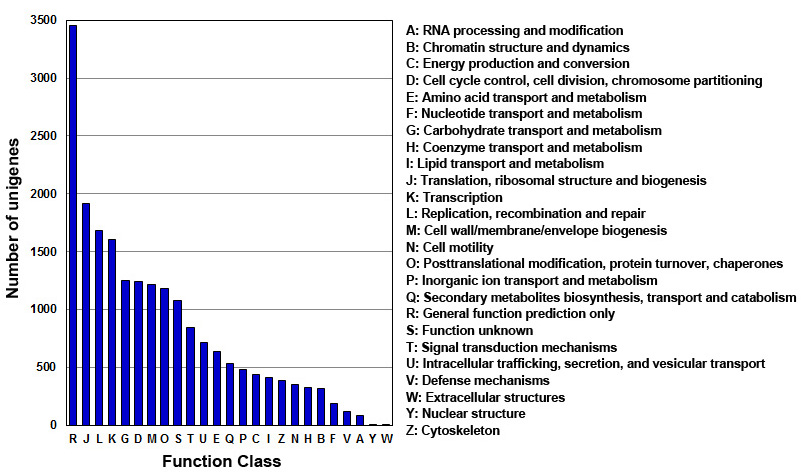


**Supplementary Figure S3. Clusters of Orthologous Groups annotation of the transcriptome.** Each letter of the x-axis represents one COG category, the y-axis shows the number of unigenes.

**Supplementary Figure S4. Evaluation and comparisons of the *de novo* assembled sequences of the four gene expression profiles** (3 individual crabs for each profile).

**
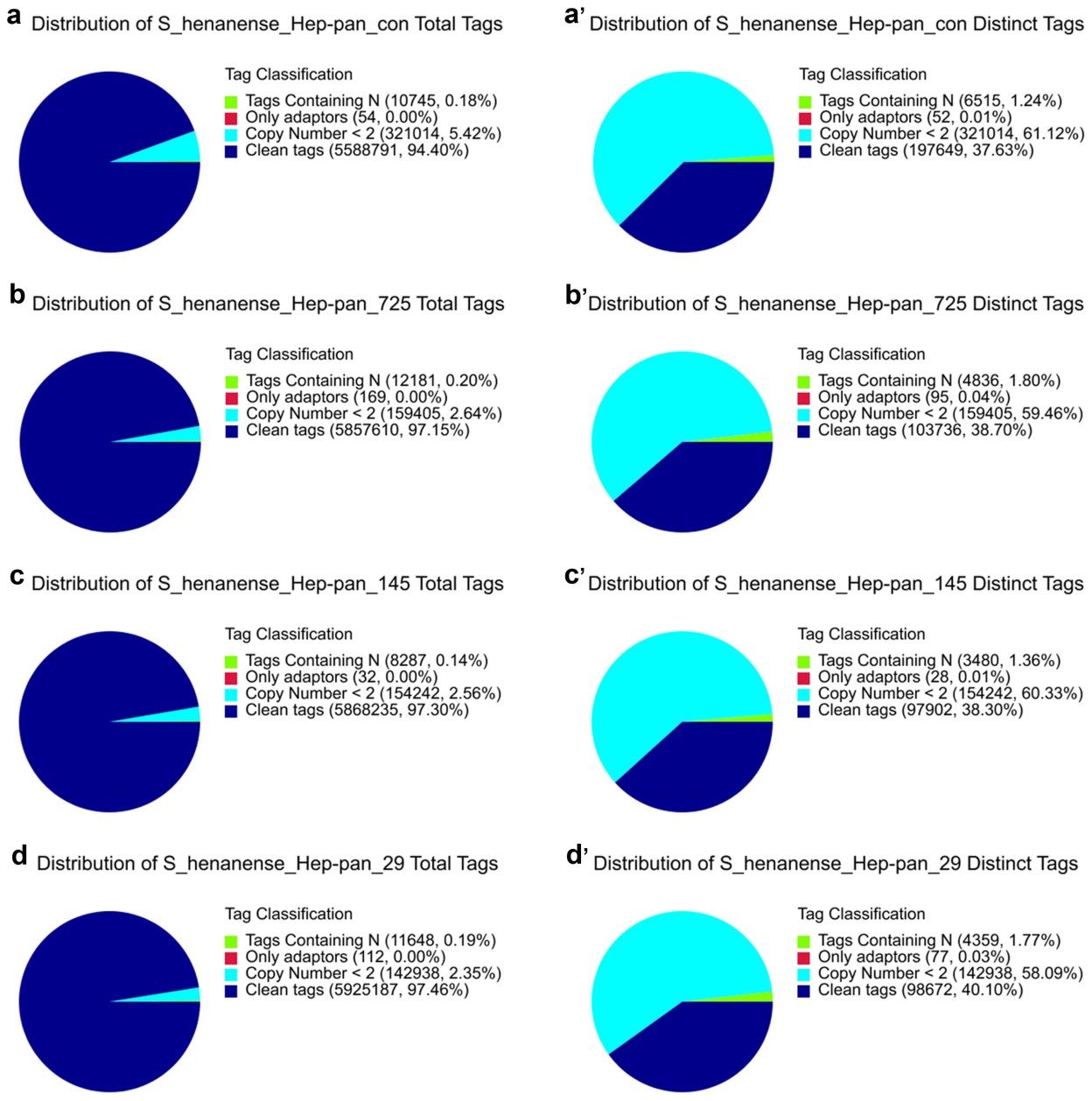
**

**(1) Assessment of sequencing quality of gene expression profiles.** The left figures show quality of the total tags. (a) control group, (b) 7.25 mg/L group, (c) 14.5 mg/L group and (d) 29.0 mg/L group. The right figures show quality of the distinct tags. (a’) control group, (b’) 7.25 mg/L group, (c’) 14.5 mg/L group and (d’) 29.0 mg/L group.

**
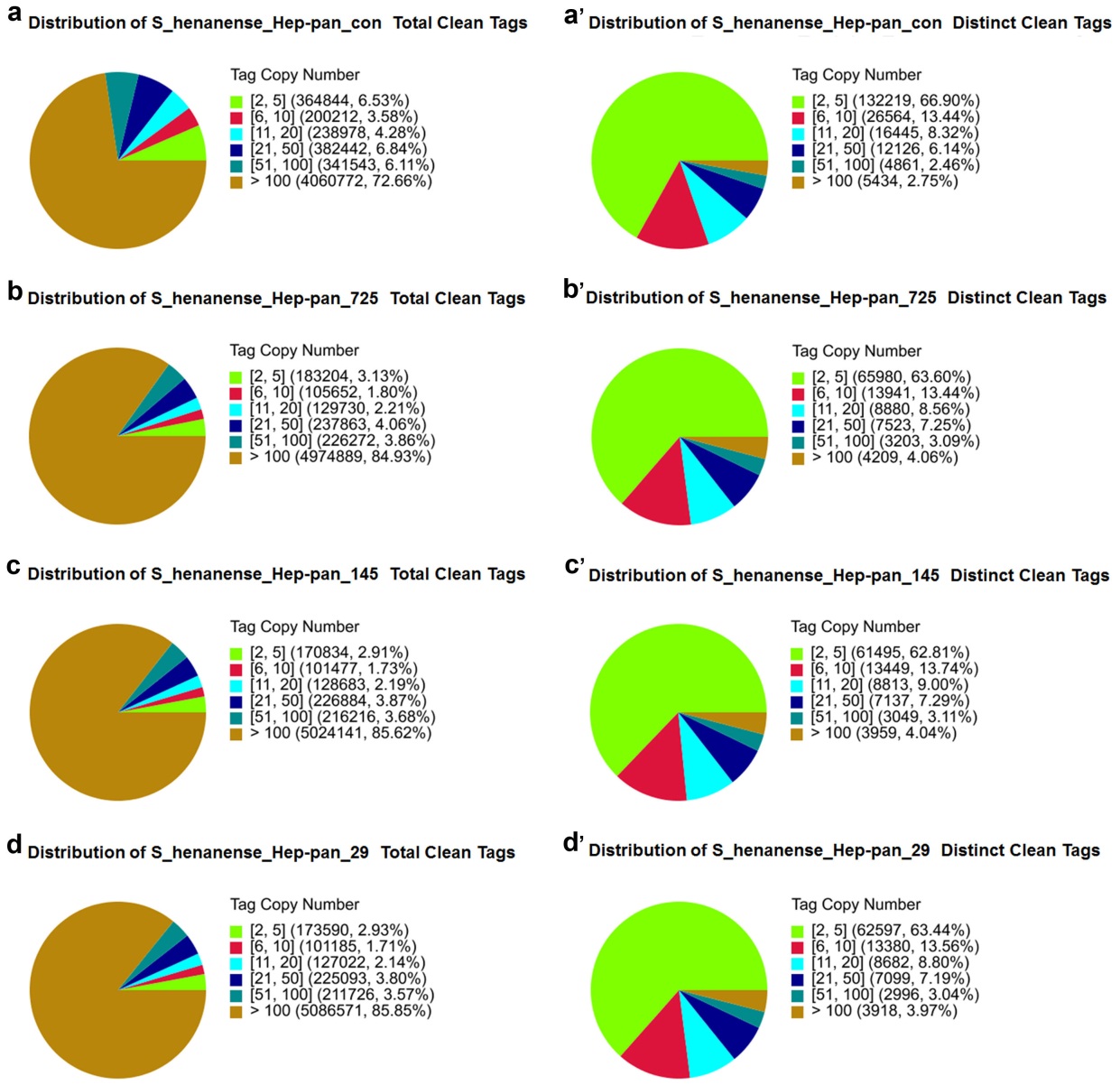
**

**(2) The distribution of the clean tag copy number.** The left shows distribution of total clean tags in control group (a), 7.25 mg/L group (b), 14.5 mg/L group (c) and 29.0 mg/L group (d). The right shows distribution of the total distinct clean tags in control group (a’), 7.25 mg/L group (b’), 14.5 mg/L group (c’) and 29.0 mg/L group (d’).


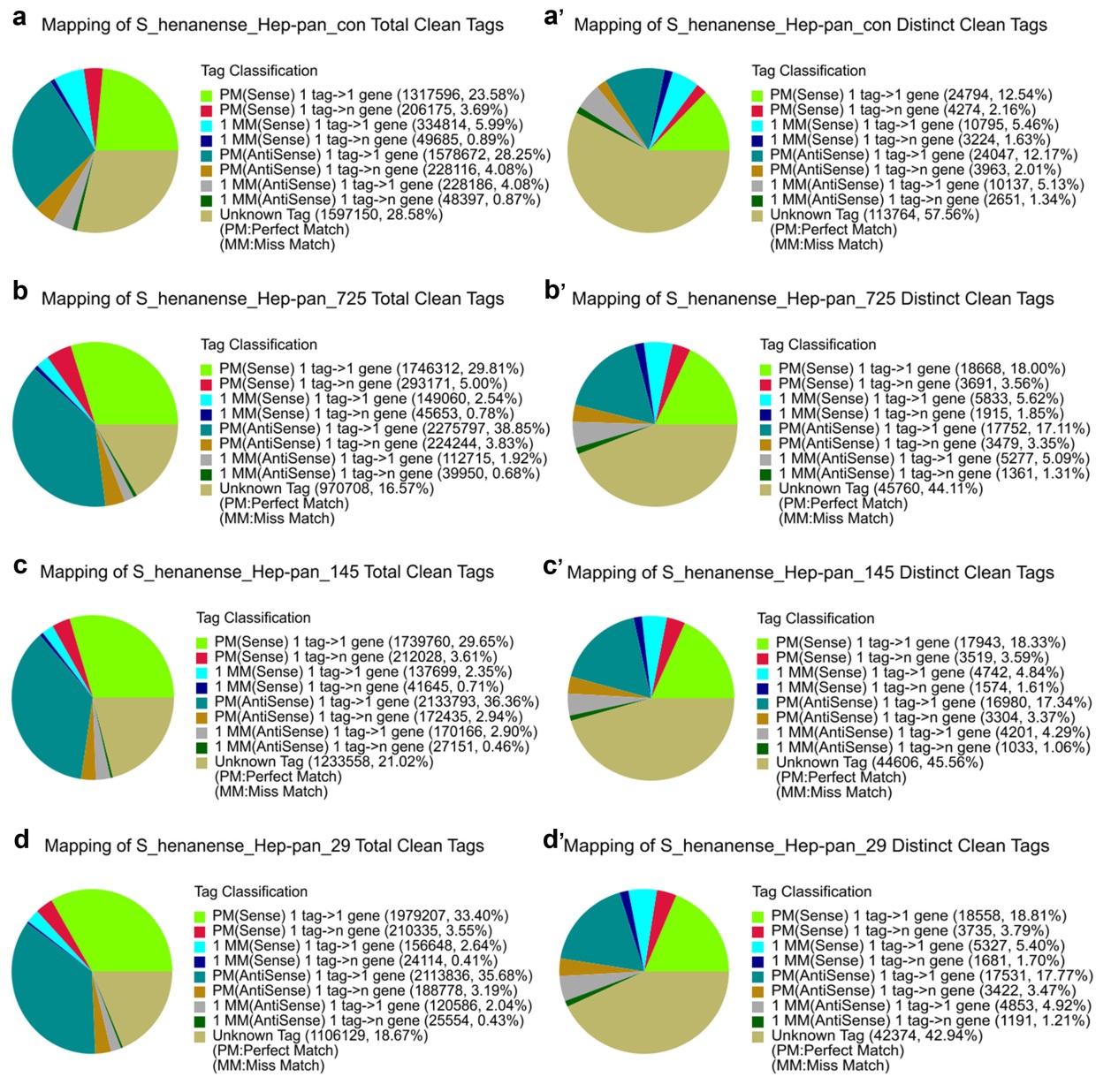


**(3) Alignment statistics of the clean tags.** The left exhibits the alignment statistics of total clean tags in control group (a), 7.25 mg/L group (b), 14.5 mg/L group (c) and 29.0 mg/L group (d). The right exhibits the alignment statistics of total distinct clean tags in control group (a’), 7.25 mg/L group (b’), 14.5 mg/L group (c’) and 29.0 mg/L group (d’).


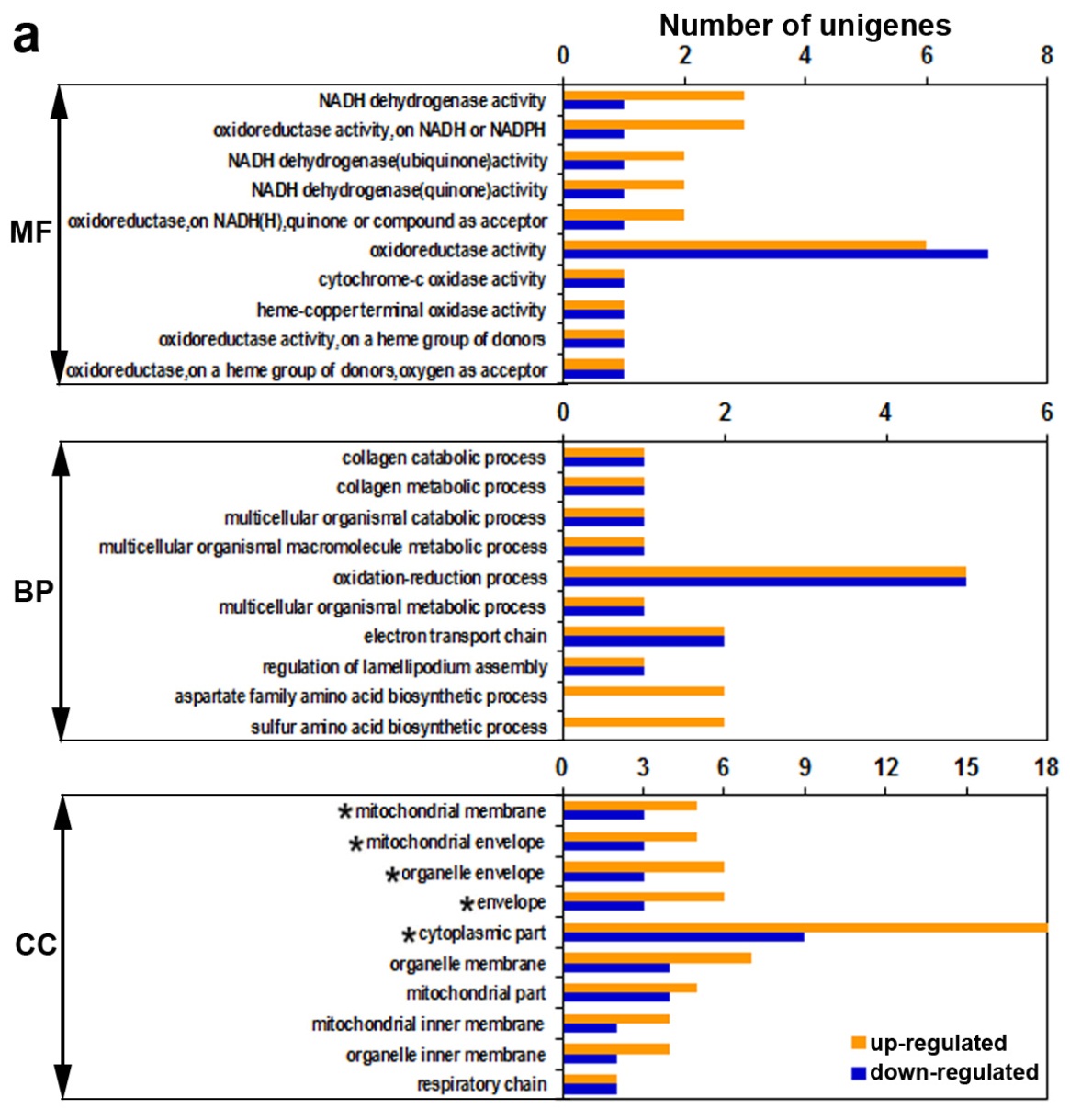


**
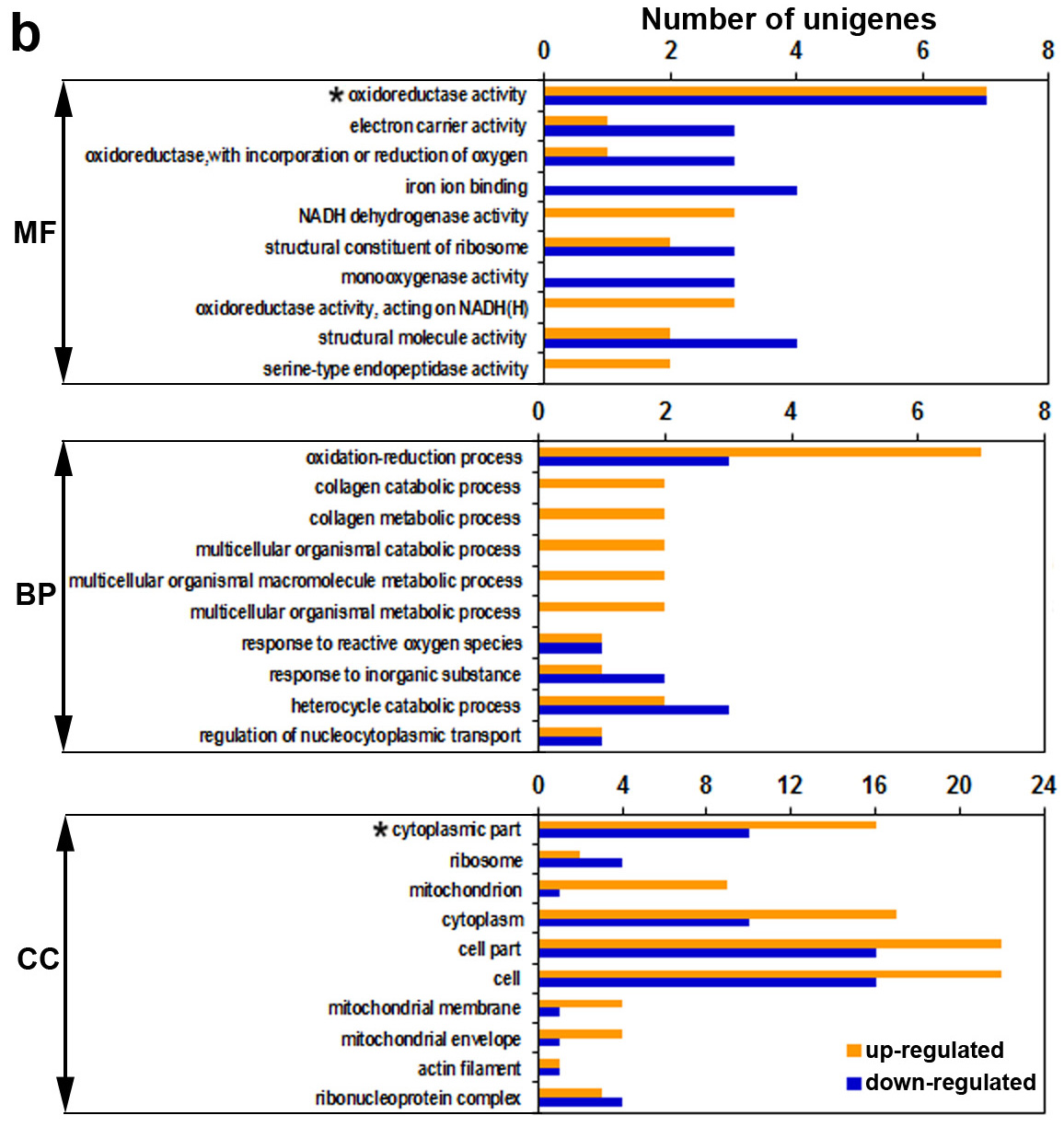
**

**
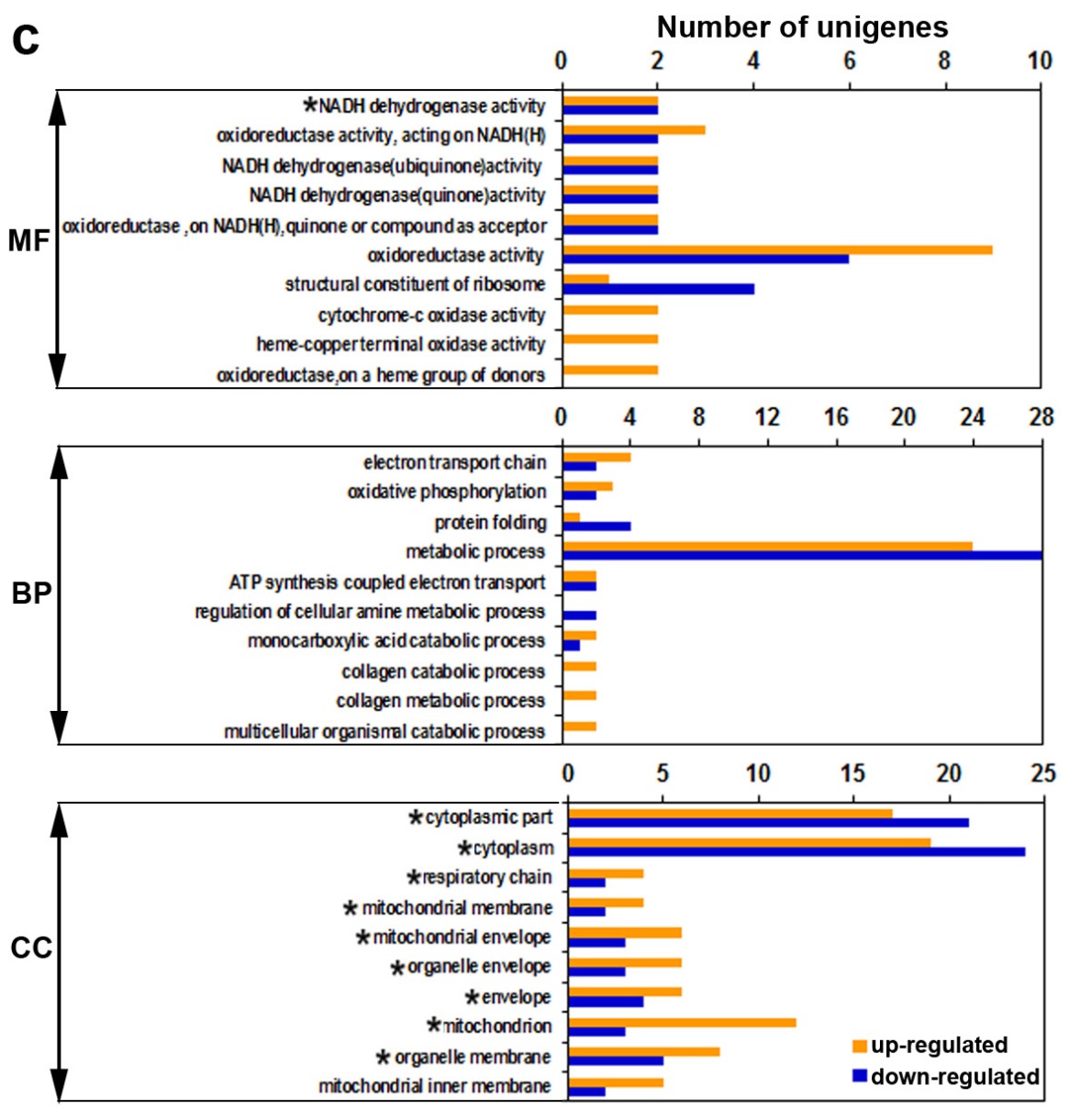
**

**Supplementary Figure S5. Up- and down-regulated unigenes of the enriched GO terms among Cd-treated groups.** Top 10 molecular functions terms, top 10 biological processes terms and top 10 cellular components terms in three panels of comparisons: (a) 7.25 mg/L vs 14.5 mg/L group, (b) 7.25 mg/L vs 29.0 mg/L group and (c) 14.5 mg/L vs 29.0 mg/L group. *****: p < 0.05.


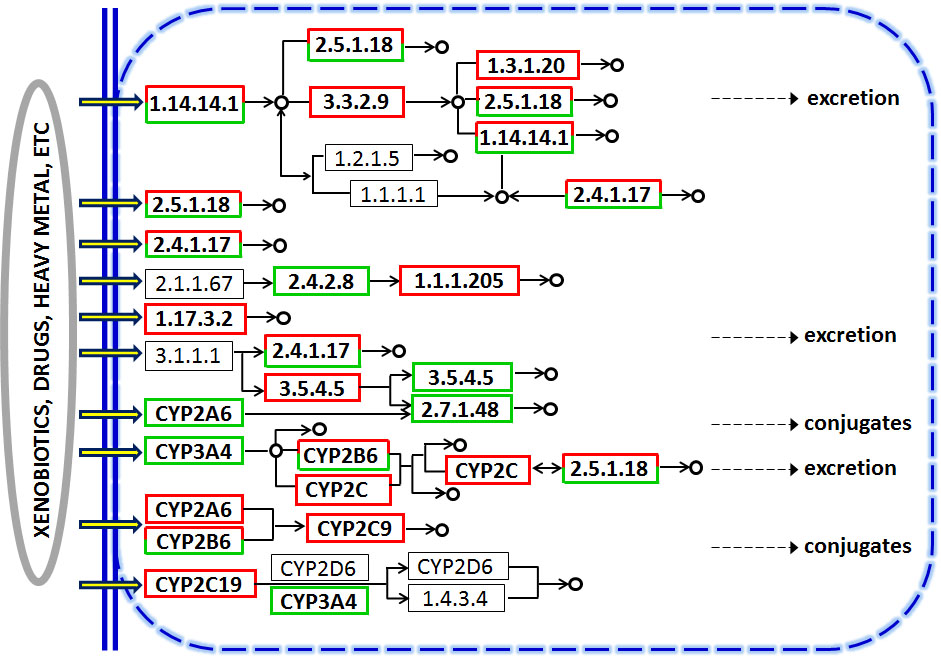


**Supplementary Figure S6. Identification of unigenes associated with the detoxification pathways in the hepatopancreas.**  Red box: up-regulated unigene(s). Green box: down-regulated unigene(s). Box with both red and green: pathway includes unigene(s) up- and down-regulated respectively.

**Supplementary Tables**

**Supplementary Table S1. Summary of gene expression profiles.** 1: control group, 2: 7.25 mg/L group, 3: 14.5 mg/L group, 4: 29.0 mg/L group (n = 3 in each group).

| Summary | 1 | 2 | 3 | 4 |
| --- | --- | --- | --- | --- |
| Raw Tags | 5920604 | 6029365 | 6030796 | 6079885 |
| Distinct Tags | 525230 | 268072 | 255652 | 246046 |
| Clean Tags | 5588791 | 5857610 | 5868235 | 5925187 |
| Distinct Clean Tags | 197649 | 103736 | 97902 | 98672 |
| Clean Tags Mapping to Genes | 3991641(71.42%) | 4886902(83.43%) | 4634677(78.98%) | 4819058(81.33%) |
| Unambiguous Tags Mapping to Genes | 3459268(61.90%) | 4283884(73.13%) | 4181418(71.26%) | 4370277(73.76%) |
| Clean Tag-mapped Genes | 29763(43.36%) | 22272(32.44%) | 21448(31.24%) | 22257(32.42%) |
| Unknown Clean Tags | 1597150(28.58%) | 970708(16.57%) | 1233558(21.02%) | 1106129(18.67%) |

**Supplementary Table S2. Altered DEGs in comparisons between each Cd-treated group and control*.* (XLS)**

**Supplementary Table S3. Altered DEGs in comparisons among the three Cd-treated groups. (XLS)**

**Supplementary Table S4. Common DEGs in comparing each Cd-treated group with control. (XLS)**

**Supplementary Table S5. Common DEGs in comparing three Cd-treated groups with each other. (XLS)**

**Supplementary Table S6. GO classification and enrichment analysis of all DEGs in comparing each Cd-treated group with control. (XLS)**

**Supplementary Table S7. GO classification and enrichment analysis of all DEGs in comparing three Cd-treated groups with each other. (XLS)**

**Supplementary Table S8. Pathway enrichment analysis of DEGs in comparisons among all four groups. (XLS)** The comparisons include: 7.25 mg/L group vs control, 14.5 mg/L group vs control, 29.0 mg/L group vs control, 7.25 mg/L group vs 14.5 mg/L group, 7.25 mg/L group vs 29.0 mg/L group and 14.5 mg/L group vs 29.0 mg/L group.

**Supplementary Table S9. Primers used for RT-qPCR in this study.** F: Forward primer sequence, R: Reverse primer sequence, Length (bp): amplicon length (base pair), E (%): amplification efficiency, R^2^: correlation factor.

| Symbol | Transcript ID | Function description | Primer sequence (5'-3'） | Length  (bp) | E (%) | R^2^ |
| --- | --- | --- | --- | --- | --- | --- |
| *acyl-CoA-BP* | Unigene21824 | acyl-CoA-binding protein | F: GACACAGCCAAATGATGAGGAGC | 130 | 97.0 | 0.9910 |
|  |  |  | R: ATTCCAGGCATCCCACTTAGC |  |  |  |
| *carboxypeptidase N* | CL1481.Contig1 | carboxypeptidase N regulatory subunit | F: CCCATTCACTTGTGGCTGTG | 129 | 98.4 | 0.9904 |
|  |  |  | R: TCACTGAACACAGCAGGGTC |  |  |  |
| *serine collagenase 1* | Unigene31174 | serine collagenase 1 precursor | F: CGTGGAAGTGGTGATGGGTG | 138 | 103.2 | 0.9990 |
|  |  |  | R: GGCAGCCTGATAATGGCAACG |  |  |  |
| *aspartate aminotransferase* | CL2118.Contig1 | aspartate aminotransferase | F: TGCTCGTGTGCTGTCTCAGG | 130 | 96.7 | 0.9999 |
|  |  |  | R: AACATCCTCCAACCACACTGC |  |  |  |
| *Hemocyanin 6* | Unigene2480 | hemocyanin subunit 6 | F: CACACCTGAAGGAACTCTCCG | 134 | 97.8 | 0.9990 |
|  |  |  | R: CGAGAACCAGTGATGCTGCTC |  |  |  |
| *Hemocyanin 1* | Unigene756 | hemocyanin subunit 1 | F: CCTCTTCAACACCCGACAACG | 141 | 96.5 | 0.9999 |
|  |  |  | R: TAGAGGGCGTAGACGAACTCG |  |  |  |
| *Alpha-L-fucosidase* | Unigene38275 | Alpha-L-fucosidase | F: TCACCAGCAAGCACCACGAG | 137 | 99.0 | 0.9994 |
|  |  |  | R:GATGTGCGATGTTTTTGCCCTG |  |  |  |
| *NADH dehydrogenase 5* | CL5396.Contig2 | NADH dehydrogenase subunit 5 | F: TTCGGTGTATGGGGTGTTTAG | 97 | 95.9 | 0.9994 |
|  |  |  | R: CCAGCTAAAAACGGAAATCCAC |  |  |  |
| *NADH dehydrogenase 1α* | Unigene25827 | NADH dehydrogenase 1 alpha subcomplex 6 | F: GGTCTGCGGCAAGTCAAGCC | 134 | 97.5 | 0.9961 |
|  |  |  | R: TCCTTGGACTTGGGTAGGTCG |  |  |  |
| *cyt c oxidase subunit II* | CL2778.Contig2 | cytochrome c oxidase subunit II | F: AAAGCCGATGCTGTACCTGGG | 108 | 96.6 | 0.9971 |
|  |  |  | R: GGAAGCTATGATTTGCCCCGC |  |  |  |
| *cyt c oxidase subunit III* | CL271.Contig1 | cytochrome c oxidase subunit III | F: GGGCTTTATTGACCTCCTCTAGG | 150 | 101.4 | 0.9938 |
|  |  |  | R: ACTTTGGGTCGCTTGGCTATGG |  |  |  |
| *HSP-B* | Unigene33763 | heat shock protein binding | F: GGCGACGAGTGCTGTTGCTG | 189 | 99.7 | 0.9986 |
|  |  |  | R: GGTGTCCCTTTCTTGCCAGG |  |  |  |
| *zinc proteinase Mpc1* | CL4129.Contig2 | zinc proteinase Mpc1 | F: CGGGTGCTGGTCGTATGTGG | 131 | 96.2 | 0.9942 |
|  |  |  | R: GGCAATGTTCGTGGTAGAAGC |  |  |  |
| *SOD* | CL2097.Contig2 | superoxide dismutase | F: GCCGACAGAAGCACACGCTG | 116 | 102.0 | 0.9999 |
|  |  |  | R: GTTGACATAGGTCTGGTGGTGC |  |  |  |
| *GST- Mu3L* | CL1026.Contig2 | glutathione S-transferase Mu 3-like | F: CTTTGACACAGGCAAGGATGC | 153 | 97.8 | 0.9979 |
|  |  |  | R: AGCAGCAGGTGTTCATCCAAC |  |  |  |
| *GST-delta* | Unigene25999 | delta glutathione S-transferase | F: TAGCACTCAACCCGCAGCAC | 123 | 98.3 | 0.9984 |
|  |  |  | R: GGAAGAGGGAGTCATCCTTGC |  |  |  |
| *MT* | CL3627.Contig1 | Metallothionein | F: CCTGTGGATGTGGAGAATCTTGC | 196 | 100.5 | 0.9978 |
|  |  |  | R: CTCACTTCATGGGGCAAGGGCTG |  |  |  |
| *selenoprotein M* | Unigene12993 | selenoprotein M | F: GCGAAGCAGTTCATCCAGGAG | 127 | 101.8 | 0.9981 |
|  |  |  | R: TGAGGTCAATCCGCTCCAGC |  |  |  |
| *C-type lectin L* | CL2649.Contig1 | C-type lectin-like | F: CAACCTCACCAATAACCAGTGCG | 125 | 98.3 | 0.9995 |
|  |  |  | R: CCTTGCCCACGGTCTTCTCG |  |  |  |
| *Lysozyme* | Unigene7865 | Lysozyme | F: AGTTGACGGACGAGTGCCTG | 134 | 94.3 | 0.9990 |
|  |  |  | R: CCAGTAAGTGATAGCCCACGG |  |  |  |
| *trypsin 1a* | CL317.Contig2 | trypsin 1a | F: ACTGCGTCGTGTCTGGCTGG | 119 | 102.9 | 0.9986 |
|  |  |  | R: TGCTTACCCCATAGGCGGCG |  |  |  |
| *cathepsin L* | Unigene30914 | cathepsin L | F: TAACTTTGGCTGTGGCGGCG | 132 | 96.8 | 0.9998 |
|  |  |  | R: GATGTCGTCGGCGTTGAAGC |  |  |  |
| *PSMB7* | Unigene33396 | proteasome subunit beta type-7-like | F: CACATCTACTCCATCCACCCAC | 118 | 98.2 | 0.9992 |
|  |  |  | R: GCGACATCTCAGGCTTCCAC |  |  |  |
| *C-type lectin receptor* | Unigene21455 | C-type lectin receptor protein | F: CTTCCCTCGCTGCCTTCGTG | 144 | 97.4 | 0.9991 |
|  |  |  | R: CATCGGCGTCCAATGCCTGAC |  |  |  |
| *chymotrypsin-L proteinase* | Unigene36462 | chymotrypsin-like proteinase | F: GCTCCCTCATCTCCAACCAG | 141 | 103.7 | 0.9980 |
|  |  |  | R: ACTGTGAAGGTTGTGCTGGTC |  |  |  |
| *Dlx6aL* | Unigene36512 | homeobox protein Dlx6a-like | F: CCTGCGAGACAGTAGGCGAG | 109 | 96.0 | 0.9994 |
|  |  |  | R: TAGGGACAGTTTGACAGCAGG |  |  |  |
| *GILT* | Unigene29024 | gamma-interferon-inducible lysosomal thiol reductase | F: TGGAGGGACAGAACTTGCTTC | 120 | 103.2 | 0.9905 |
|  |  |  | R: ACTCACCCACCTGGTCCTCG |  |  |  |
| *18S rRNA* | CL2184.Contig2 | 18S ribosomal RNA | F: CAGACAAATCGCTCCACCAAC | 121 | 102.3 | 0.9992 |
|  |  |  | R: GACTCAACACGGGGAACCTCA |  |  |  |
| *Rpl38* | Unigene21407 | 60S ribosomal protein L38 | F: GTTAGACGGTGACTGCTGCTC | 104 | 101.1 | 0.9992 |
|  |  |  | R: TCTTCACCGACTTTGCGTCC |  |  |  |
| *GADPH* | Unigene14812 | Glyceraldehyde-3-phosphate dehydrogenase | F: TCATCTCTGCCCCCTCTGCTG | 198 | 95.4 | 0.9976 |
|  |  |  | R: CTGGGTGGCAGTGATGGCATG |  |  |  |
| *Rpl13* | Unigene21717 | 60S ribosomal protein L 13 | F: TACCACCGCACCAACAAGTC | 104 | 103.8 | 0.9943 |
|  |  |  | R: GCCTTGTTCGCCTTCAGTGG |  |  |  |
| *Rpl44* | Unigene21240 | ribosomal protein L44e | F: CTGGAAGTGCAAGAAGCACCAG | 110 | 97.9 | 0.9999 |
|  |  |  | R: CATACCCAGACTGCTTCCTGTCG |  |  |  |
